# Torque Accuracy of Spring‐Style Dental Torque Wrenches After Repeated Mechanical Loading and Sterilization: An In Vitro Study

**DOI:** 10.1155/ijod/4941557

**Published:** 2026-08-24

**Authors:** Batuhan Aydın, Zehra Gülerol Aydın, Serkan Polat

**Affiliations:** ^1^ Department of Oral and Maxillofacial Surgery, Faculty of Dentistry, Bolu Abant İzzet Baysal University, Bolu, Türkiye, ibu.edu.tr

**Keywords:** dental abutments, dental implants, insertion torque, mechanical torque-limiting device, torque wrench

## Abstract

This in vitro study evaluated the torque accuracy of spring‐style mechanical torque‐limiting devices (MTLDs) from three manufacturers under clinically simulated conditions. Nine devices (Implance, Astra Tech, and DIO) were tested with a digital torque tester. Target torque values were 25 and 35 Ncm for Implance and Astra Tech and 20 and 35 Ncm for DIO. Each device underwent 40 experimental cycles, including baseline measurement, mechanical fatigue by 20 loading repetitions at target torque, post‐fatigue measurement, and steam sterilization. Torque difference and percentage deviation from target value were calculated; deviations exceeding ±10% were classified as outside the acceptable range. Torque difference and percentage deviation were analyzed using aligned rank transform (ART) mixed ANOVA with Holm‐adjusted comparisons. The experimental cycle significantly affected torque difference in all manufacturers, whereas the measurement stage did not. Percentage deviation was significantly associated with experimental cycle, manufacturer, and their interaction. Overall median percentage deviation was 11.43% for Implance, 3.33% for Astra Tech, and 16.67% for DIO. Torque accuracy was manufacturer‐dependent and changed over repeated experimental cycles comprising mechanical loading and sterilization, supporting verification before clinical use and periodic recalibration of spring‐style MTLDs.

## 1. Introduction

Primary implant stability is an important requirement for successful osseointegration and can be evaluated using objective parameters such as insertion torque value (ITV), implant stability quotient (ISQ) obtained by resonance frequency analysis, and periotest value (PTV) measured with a Periotest device [1–3]. Compared with other stability assessment methods, ITV is more practical and time‐efficient because it does not require additional equipment and can be applied immediately during surgery [4, 5].

Dental implant manufacturers provide mechanical torque‐limiting devices (MTLDs) to help clinicians deliver the recommended torque during implant placement and prosthetic screw tightening [6, 7]. Dental torque devices include electronic and mechanical systems; mechanical designs are commonly classified as spring‐style (beam‐type) or friction‐style/toggle‐type wrenches [8–10]. Spring‐style MTLDs operate through elastic beam deflection against a graduated scale, allowing different torque values to be applied with the same instrument [8, 9]. Although this adjustability is clinically practical, the delivered torque may be affected by scale reading, operator handling, device condition, and repeated clinical use [9, 10]. Previous studies have reported that spring‐style devices may show greater accuracy than friction‐style counterparts, although performance varies according to the manufacturer and testing protocol [8–10].

Previous studies have shown that the torque output of MTLDs may change after sterilization and repeated use, although the reported effects vary according to the device design and testing protocol [10–13]. Fayaz et al. [11], for example, found that friction‐style MTLDs remained within the ±10% tolerance range after the first sterilization cycle, but deviations became more variable with subsequent cycles. Although those findings cannot be directly extrapolated to spring‐style devices, they support the need to monitor torque accuracy throughout the clinical service life of MTLDs.

Accurate torque delivery is clinically relevant in both surgical and prosthetic implant procedures. During implant placement, insertion torque is commonly used as an indicator of primary stability, particularly in immediate‐loading protocols [14]. In the prosthetic phase, the applied tightening torque determines the preload generated within the implant‐abutment screw joint [7, 15, 16]. Insufficient torque may contribute to screw loosening, preload loss, fracture, and prosthetic complications, whereas excessive torque may deform the screw, damage the threads, or compromise implant components [17–21]. Therefore, the torque delivered by an MTLD should correspond closely to the manufacturer‐recommended value for the specific implant‐abutment system [6, 22, 23].

Most previous studies assessed MTLD accuracy by testing the wrench directly with a torque gauge [6, 8, 24, 25]. In the present study, each spring‐style MTLD was evaluated on the corresponding implant system from its respective manufacturer, rather than as an isolated wrench, and underwent repeated mechanical loading and steam sterilization under a standardized experimental protocol.

Available studies differ considerably in device type, testing setup, repeated‐use simulation, sterilization protocol, and operator‐related factors, making direct comparison difficult and limiting the generalizability of existing findings [26]. These differences support the use of standardized experimental models in which MTLDs are tested with their corresponding implant systems rather than as isolated devices.

Therefore, this study evaluated the torque accuracy of spring‐style MTLDs from three manufacturers under simulated clinical‐use conditions after repeated mechanical loading and steam sterilization, with the aim of informing verification and recalibration strategies.

## 2. Materials and Methods

### 2.1. Devices and Study Design

**Figure 1:**
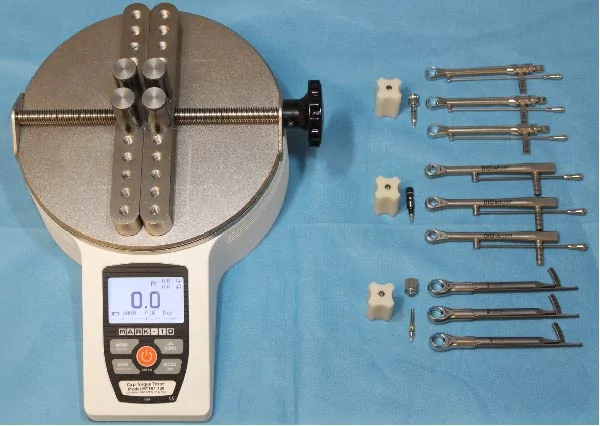
Digital torque tester; manufacturer‐specific spring‐style MTLDs; and manufacturer‐specific implants inserted in resin blocks.

**Figure 2:**
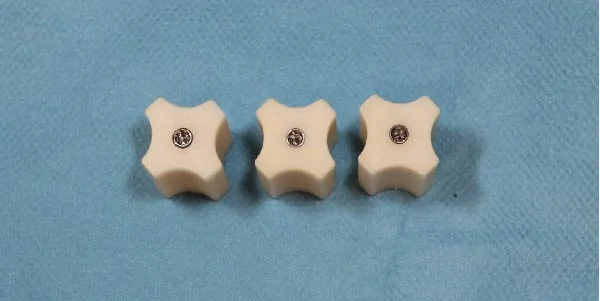
Insertion of manufacturer‐specific implants into CAD/CAM‐fabricated resin blocks at the simulated crestal bone level.

**Figure 3:**
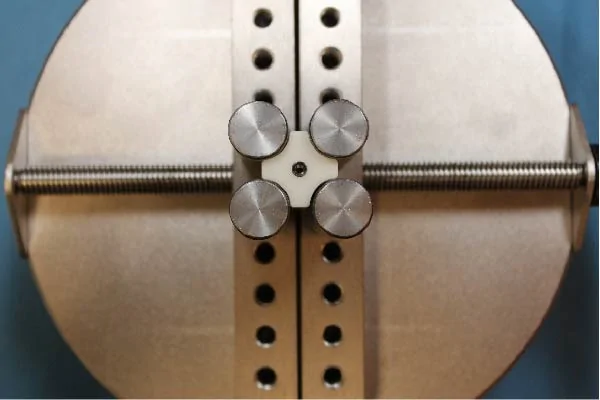
Placement of implants inserted into resin blocks on the measuring platform of the digital torque tester.

Nine new and unused spring‐style MTLDs were evaluated, with three devices from each manufacturer: Implance (AGS Medikal, İstanbul, Turkey), Astra Tech Implant System (Dentsply Sirona Implants, Mölndal, Sweden), and DIO Implant (DIO Corporation, Busan, Republic of Korea) (Figure 1). A unique identification number was assigned to each MTLD. Manufacturer‐specific implants were inserted into CAD/CAM‐fabricated resin blocks, with the implant platforms positioned at the simulated crestal bone level (Implance: 4.3 × 10 mm; Astra Tech: 4.2 × 11 mm; and DIO: 4.0 × 10 mm) (Figures 2 and 3). Each MTLD was used with the manufacturer‐specific mounting component (e.g., mount driver or mount adapter) supplied for the corresponding implant system.

**Figure 4:**
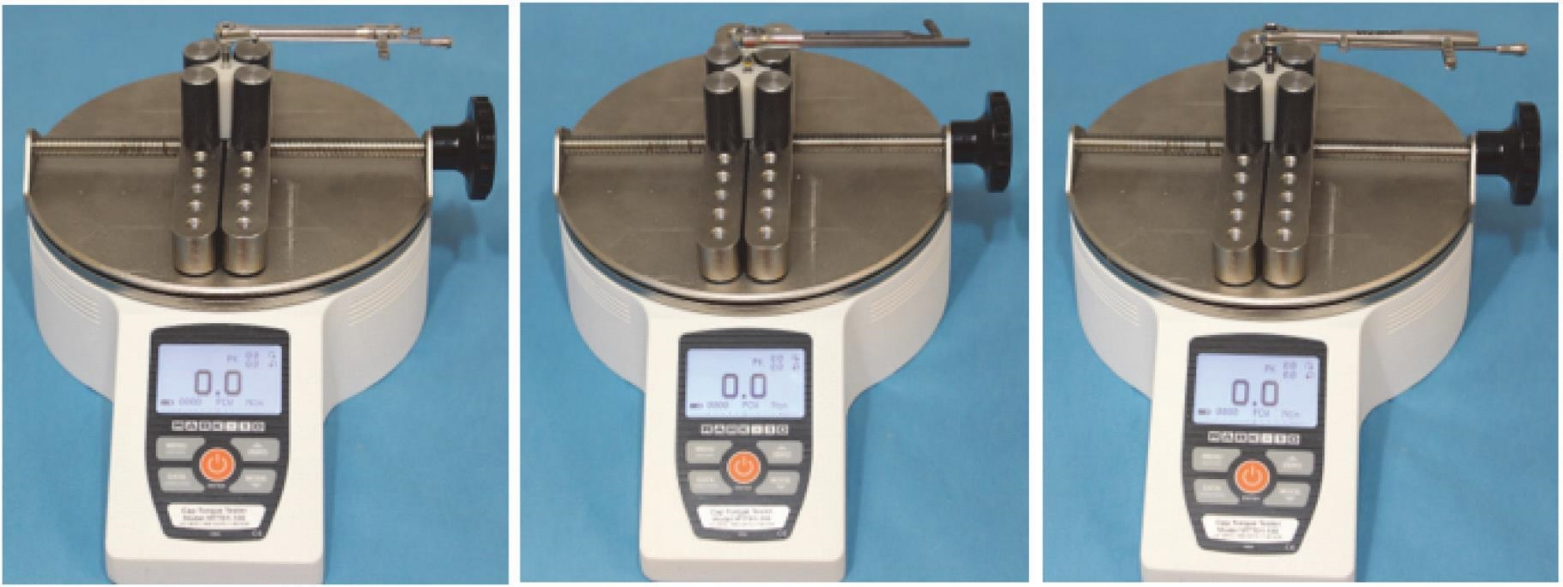
Experimental setup showing the connection of the manufacturer‐specific implant systems to the corresponding spring‐style MTLDs on the measuring platform of the digital torque tester.

**Figure 5:**
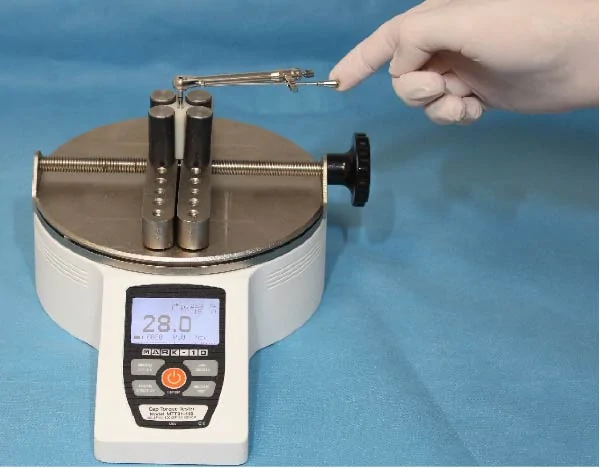
Torque measurement using the digital torque tester.

Measurements were performed after each MTLD was connected to the implant system corresponding to its manufacturer (Figure 4). To minimize operator‐dependent variability, all measurements were performed by a single investigator. Force was applied in a horizontal plane using the distal phalanx of the index finger at a predefined grip point on the handle, applied slowly and steadily in a clockwise direction until the indicator rod aligned with the target mark on the built‐in scale. To minimize parallax error, the scale was read at a perpendicular angle (90°). Torque output was verified using a calibrated digital torque tester (Mark‐10 Cap Torque Tester, model MTT01‐100, USA) with an accuracy ±0.2% of full scale (Figure 5).

For MTLDs without a factory‐etched zero reference, a standardized zero mark was created at the baseline by a single operator using a fine rotary instrument. This mark was used to read the scale consistently during subsequent measurements and to check whether the indicator returned to the zero reference after repeated cycling. Before each measurement set, the wrench was inspected under no‐load conditions. The failure of the indicator to return to the zero reference was predefined as an exclusion criterion suggestive of possible plastic deformation. The zero reference mark was only a visual guide and did not modify any functional component or the mechanism of the device.

Target torque values were 25 and 35 Ncm for Implance and Astra Tech and 20 and 35 Ncm for DIO. The 35 Ncm value was used to represent a clinically relevant implant placement torque, whereas the 25 Ncm torque value represented prosthetic abutment screw tightening [14, 27–29]. When 25 Ncm was not available on the MTLD scale, the closest marked value was used; therefore, 20 Ncm was selected for DIO. Before each measurement, the digital torque tester was set to the peak mode and zeroed.

Cycle 0 was defined as the first assessment performed immediately after the new and unused MTLDs were removed from their original packaging before any autoclave sterilization within the study protocol. Baseline torque measurements were first recorded. Each device then underwent mechanical loading consisting of 10 consecutive loading repetitions at each target torque setting: 25 and 35 Ncm for Implance and Astra Tech and 20 and 35 Ncm for DIO. Post‐fatigue measurements were then recorded, completing the cycle 0 assessment. After cycle 0 measurements, the devices were sterilized in an autoclave. Each subsequent experimental cycle followed the same sequence: baseline measurement, mechanical loading, post‐fatigue measurement, and autoclave sterilization. This protocol was repeated for 40 experimental cycles per MTLD. At each measurement stage, the torque output at the designated torque settings was measured three times, and the mean value was used for analysis.

After completion of each measurement stage, each MTLD underwent steam sterilization in an autoclave (Kronos B23; Newmed, Quattro Castella, Italy) at 134°C under 2.1 bar for 5 min. One experimental cycle was defined as mechanical loading, followed by sterilization, and this cycle was repeated 40 times for each device. Before each sterilization procedure, the identification number of each device was marked on the sterilization pouch.

### 2.2. Sample Size Calculation

Sample size calculation was performed using 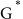
Power 3.1.9.2 software [30]. The mechanical torque‐limiting device (MTLD) was considered an independent experimental unit. Because no directly comparable preliminary data were available, a large standardized effect size of *f* = 0.40 was assumed based on Cohen’s convention for ANOVA designs [31]. For the repeated measures within–between interaction model, the number of groups was set to 3, and the number of repeated measurements was set to 10, corresponding to five cycle levels (0, 10, 20, 30, and 40) assessed at two time points (baseline and post‐fatigue). Assuming a correlation of 0.50 among repeated measurements, with α = 0.05% and 80% power, the minimum required sample size was calculated as nine devices, corresponding to three devices per manufacturer.

### 2.3. Outcome Measures

Torque difference was calculated for each measurement stage (baseline and post‐fatigue) as |(T_measured − T_target)|, where T_measured is the torque measured by the digital torque tester and T_target is the target torque indicated on the MTLD. This outcome was used for within each manufacturer comparisons across experimental cycles, measurement stages, and target torque values. In addition, percentage deviation from the target torque value was calculated, and this outcome was used for between each manufacturer comparisons of MTLD performance across the three manufacturers. Percentage deviation (PerDev, %) was calculated for each measurement as |(T_measured − T_target)|/T_target × 100, where T_measured is the torque measured by the digital tester and T_target is the target torque indicated on the MTLD. Measurements were recorded for all 40 cycles; for statistical analysis, cycles 0, 10, 20, 30, and 40 were selected a priori.

### 2.4. Statistical Analysis

The torque difference and percentage deviation from the target torque were analyzed as dependent variables to evaluate calibration accuracy over repeated cycling. Analyses were performed in R (v4.4.1; R Foundation for Statistical Computing, Vienna, Austria). Normality was assessed using the Shapiro–Wilk test. Because outcomes were not normally distributed, an aligned rank transform (ART) mixed ANOVA was used. For torque difference, analyses were conducted within each manufacturer, with experimental cycle (0, 10, 20, 30, and 40), measurement stage (baseline vs. post‐fatigue), and target torque values (25 and 35 Ncm for Implance and Astra Tech; 20 and 35 Ncm for DIO) specified as fixed effects, and device ID included as a random (grouping) factor to account for repeated measurements. For between‐manufacturer comparisons, percentage deviation was analyzed in a model including the manufacturer, experimental cycle, and their interaction, with device ID as a random factor. Post hoc comparisons were performed using estimated marginal means with Holm correction. Effect sizes were reported as partial eta‐squared (ηp^2^). Within each manufacturer, the consistency of repeated measurements was assessed using ICC(3,1) (two‐way mixed‐effects, consistency, and single‐measure). The association between the manufacturer and exceedance of the ±10% tolerance range was examined using Pearson’s chi‐square test; significant results were followed by pairwise column‐proportion Z tests with Bonferroni correction. ART analyses were conducted using ARTool and post hoc comparisons using emmeans. Because the quantitative variables were not normally distributed, they are presented as the median (minimum–maximum). Statistical significance was set at *p* < 0.05.

## 3. Results

None of the MTLDs exhibited failure to return to the predefined zero reference under no‐load conditions at any measurement point; accordingly, no device met the exclusion criterion suggestive of plastic deformation or permanent set during repeated cycling.

In the Implance group, torque difference varied across experimental cycles (*p* = 0.004). Post hoc analysis showed higher values at cycle 30 than at cycles 10 and 40, while the remaining cycle comparisons were not statistically different. Neither measurement stage nor target torque value was associated with torque difference (*p* = 0.311 and *p* = 0.088, respectively). No significant two‐ or three‐way interactions were detected (all *p*  > 0.05). Detailed descriptive and post hoc results are presented in Tables 1 and 2.

**Table 1:** Comparison of torque difference values according to experimental cycle, target torque values, and measurement stage (Implance).

Effect	Test statistics	*p*	ES
Experimental cycle	4.697	0.004	0.320
Measurement stage	1.053	0.311	0.026
Target torque values	3.051	0.088	0.071
Experimental cycle × measurement stage	0.409	0.800	0.039
Experimental cycle × target torque values	0.555	0.696	0.053
Measurement stage × target torque values	0.042	0.838	0.001
Experimental cycle × measurement stage x target torque values	0.623	0.649	0.059

*Note:* ART mixed ANOVA; *p* < 0.05 was considered statistically significant.

Abbreviation: ES, effect size (partial eta‐squared).

**Table 2:** Descriptive statistics and multiple comparisons for torque difference values according to experimental cycle, target torque values, and measurement stage (Implance).

Measurement stage	Target torque values (Ncm)	Experimental cycle					Overall across experimental cycles
		Cycle 0	Cycle 10	Cycle 20	Cycle 30	Cycle 40	
Baseline	25	3.5 (3–4.5)	3.17 (3–4.17)	3.67 (3.33–3.83)	3.83 (3.83–5)	3.33 (2.33–3.5)	3.5 (2.33–5)
	35	3 (2–5)	3.17 (1.83–4.5)	2.67 (2.5–3.33)	3.83 (3.83–4)	3 (2.33–4)	3.17 (1.83–5)
	Overall	3.25 (2–5)	3.17 (1.83–4.5)	3.33 (2.5–3.83)	3.83 (3.83–5)	3.17 (2.33–4)	3.42 (1.83–5)

Post‐fatigue	25	2.5 (2.5–3.5)	3.17 (2.83–4.5)	3.67 (3.17–4)	4 (3.5–4.5)	3 (2.33–3.17)	3.17 (2.33–4.5)
	35	3.5 (2.5–5)	2.67 (2–3)	3.17 (2.67–3.67)	3.67 (3.17–4)	2.67 (2–2.83)	3 (2–5)
	Overall	3 (2.5–5)	2.92 (2–4.5)	3.42 (2.67–4)	3.83 (3.17–4.5)	2.75 (2–3.17)	3.17 (2–5)

Overall across measurement stages	25	3.25 (2.5–4.5)	3.17 (2.83–4.5)	3.67 (3.17–4)	3.92 (3.5–5)	3.08 (2.33–3.5)	3.5 (2.33–5)
	35	3.25 (2–5)	2.83 (1.83–4.5)	2.92 (2.5–3.67)	3.83 (3.17–4)	2.75 (2–4)	3.08 (1.83–5)
	Overall across target torque values	3.25 (2–5)^ab^	3.08 (1.83–4.5)^a^	3.33 (2.5–4)^ab^	3.83 (3.17–5)^b^	2.92 (2–4)^a^	

*Note:* Median (minimum–maximum).

^a,b^Experimental cycles that share the same letter are not significantly different.

In the Astra Tech group, torque difference varied across experimental cycles (*p*  < 0.001). Post hoc analysis showed higher values at cycle 0 than at all later cycles. Measurement stage was not associated with torque difference (*p* = 0.059), whereas target torque value was related to torque difference (*p*  < 0.001), with higher torque difference values at 35 Ncm than at 25 Ncm. No significant two‐ or three‐way interactions were detected (all *p*  > 0.05). Detailed descriptive and pairwise comparison results are presented in Tables 3 and 4.

**Table 3:** Comparison of torque difference values according to experimental cycle, target torque values, and measurement stage (Astra Tech).

Effect	Test Statistics	*p*	ES
Experimental cycle	17.344	<0.001	0.634
Measurement stage	3.790	0.059	0.086
Target torque values	13.459	<0.001	0.252
Experimental cycle × measurement stage	1.020	0.409	0.093
Experimental cycle × target torque values	0.966	0.437	0.088
Measurement stage × target torque values	3.740	0.061	0.086
Experimental cycle × measurement stage × target torque values	0.426	0.789	0.040

*Note:* ART mixed ANOVA; *p*  < 0.05 was considered statistically significant.

Abbreviation: ES, effect size (partial eta‐squared).

**Table 4:** Descriptive statistics and multiple comparisons for torque difference values according to experimental cycle, target torque values, and measurement stage (Astra Tech).

Measurement stage	Target torque values (Ncm)	Experimental cycle					Overall across experimental cycles
		Cycle 0	Cycle 10	Cycle 20	Cycle 30	Cycle 40	
Baseline	25	2.5 (2–3.5)	1.33 (0.67–2.17)	0.83 (0.67–1)	1 (0.17–1.33)	0.67 (0–0.67)	1 (0–3.5)
	35	3 (2–4)	1.33 (0.17–2.67)	1 (0.83–1)	1 (1–1.33)	1 (0.33–1.5)	1 (0.17–4)
	Overall	2.75 (2–4)	1.33 (0.17–2.67)	0.92 (0.67–1)	1 (0.17–1.33)	0.67 (0–1.5)	1 (0–4)

Post‐fatigue	25	2 (0.5–3)	0.83 (0.67–2)	0.33 (0–1.33)	0.33 (0.17–1)	0.17 (0–0.33)	0.5 (0–3)
	35	2.5 (2.5–2.5)	1 (1–2.17)	1.17 (0.17–1.17)	1.5 (1.5–1.67)	0.83 (0.83–1.33)	1.33 (0.17–2.5)
	Overall	2.5 (0.5–3)	1 (0.67–2.17)	0.75 (0–1.33)	1.25 (0.17–1.67)	0.58 (0–1.33)	1 (0–3)

Overall across measurement stages	25	2.25 (0.5–3.5)	1.08 (0.67–2.17)	0.75 (0–1.33)	0.67 (0.17–1.33)	0.25 (0–0.67)	0.75 (0–3.5)
	35	2.5 (2–4)	1.17 (0.17–2.67)	1 (0.17–1.17)	1.42 (1–1.67)	0.92 (0.33–1.5)	1.25 (0.17–4)
	Overall across target torque values	2.5 (0.5–4)^a^	1.17 (0.17–2.67)^b^	0.92 (0–1.33)^cd^	1 (0.17–1.67)^bc^	0.67 (0–1.5)^d^	

*Note:* Median (minimum–maximum).

^a–d^Experimental cycles that share the same letter are not significantly different.

In the DIO group, torque difference varied across experimental cycles (*p* = 0.007). Post hoc analysis showed higher values at cycles 10 and 30 than at cycle 40, while the remaining cycle comparisons were not statistically different. Measurement stage was not associated with torque difference (*p* = 0.243). In contrast, target torque value was strongly related to torque difference (*p* < 0.001), with higher torque difference values at 35 Ncm than at 20 Ncm. No significant two‐ or three‐way interactions were detected (all *p*  > 0.05). Detailed descriptive and post hoc results are presented in Tables 5 and 6.

**Table 5:** Comparison of torque difference values according to experimental cycle, target torque values, and measurement stage (DIO).

Effect	Test statistics	*p*	ES
Experimental cycle	4.155	0.007	0.294
Measurement stage	1.402	0.243	0.034
Target torque values	316.580	<0.001	0.888
Experimental cycle × measurement stage	0.878	0.486	0.081
Experimental cycle × target torque values	1.197	0.328	0.107
Measurement stage × target torque values	1.188	0.283	0.029
Experimental cycle × measurement stage × target torque values	0.350	0.843	0.034

*Note:* ART mixed ANOVA; *p*  < 0.05 was considered statistically significant.

Abbreviation: ES, effect size (partial eta‐squared).

**Table 6:** Descriptive statistics and multiple comparisons for torque difference values according to experimental cycle, target torque values, and measurement stage (DIO).

Measurement stage	Target torque values (Ncm)	Experimental cycle					Overall across experimental cycles
		Cycle 0	Cycle 10	Cycle 20	Cycle 30	Cycle 40	
Baseline	20	3.5 (1.5–4)	3.17 (3–3.83)	3.83 (2.33–4)	3 (3–3.67)	2.5 (2.5–3.5)	3.17 (1.5–4)
	35	6.5 (4.5–7)	6.17 (5.5–8)	6.33 (4.67–6.33)	7 (5.5–7.67)	6.33 (6.33–6.5)	6.33 (4.5–8)
	Overall	4.25 (1.5–7)	4.67 (3–8)	4.34 (2.33–6.33)	4.59 (3–7.67)	4.92 (2.5–6.5)	4.25 (1.5–8)

Post‐fatigue	20	4 (1.5–4)	3.5 (3–4.67)	3 (1.67–3.33)	3.17 (2.5–4.17)	2.83 (2.17–3)	3 (1.5–4.67)
	35	5.5 (5–7)	6.5 (5.5–7.83)	5.33 (4.83–5.83)	6.83 (5.67–7.17)	5.67 (5.17–5.83)	5.67 (4.83–7.83)
	Overall	4.5 (1.5–7)	5.09 (3–7.83)	4.08 (1.67–5.83)	4.92 (2.5–7.17)	4.09 (2.17–5.83)	4.75 (1.5–7.83)

Overall across measurement stages	20	3.75 (1.5–4)	3.34 (3–4.67)	3.17 (1.67–4)	3.09 (2.5–4.17)	2.67 (2.17–3.5)	3.09 (1.5–4.67)
	35	6 (4.5–7)	6.34 (5.5–8)	5.58 (4.67–6.33)	6.92 (5.5–7.67)	6.08 (5.17–6.5)	6.25 (4.5–8)
	Overall across target torque values	4.25 (1.5–7)^ab^	5.09 (3–8)^a^	4.34 (1.67–6.33)^ab^	4.84 (2.5–7.67)^a^	4.34 (2.17–6.5)^b^	

*Note:* Median (minimum–maximum).

^a,b^Experimental cycles that share the same letter are not significantly different.

**Figure 6:**
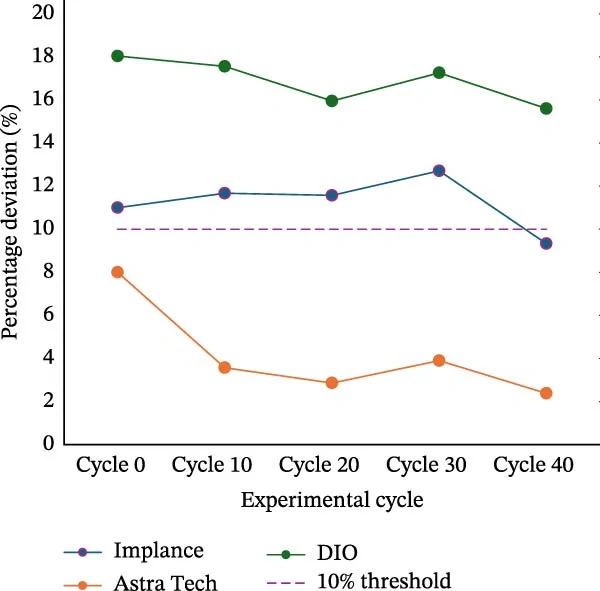
Median percentage deviation from the target torque value by manufacturer across experimental cycles.

**Figure 7:**
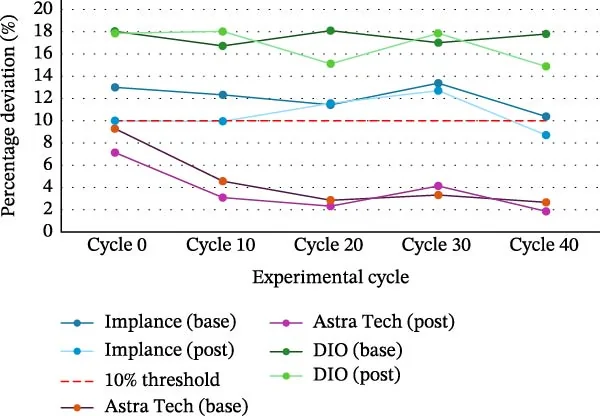
Percentage deviation values by experimental cycle, measurement stage, and manufacturer.

Percentage deviation changed across experimental cycles (*p*  < 0.001) and differed among manufacturers (*p*  < 0.001). Measurement stage was not statistically associated with percentage deviation (*p* = 0.081). Pairwise comparisons showed differences between cycles 0 and 20, cycles 0 and 40, cycles 10 and 40, and cycles 30 and 40. Among manufacturers, Astra Tech had the lowest overall deviation and DIO had the highest, with pairwise comparisons confirming differences among all three manufacturers. The cycle × manufacturer interaction was significant (*p*  < 0.001), indicating that the effect of experimental cycle on percentage deviation was not the same across manufacturers. The remaining interactions were not statistically significant: cycle × measurement stage (*p* = 0.878), measurement stage × manufacturer (*p* = 0.943), and the three‐way interaction (*p* = 0.870). These patterns are illustrated in Figures 6 and 7.

There was a statistically significant association between manufacturer and exceedance of the ±10% tolerance range (*p*  < 0.001). The exceedance rates were 60% for Implance, 5% for Astra Tech, and 95% for DIO. Pairwise comparisons indicated significant differences among all manufacturers (Table 7).

**Table 7:** Association between manufacturer and exceedance of the ±10% tolerance range.

±10% Tolerance exceedance status	Manufacturer			Overall	Test statistics	*p*
	Implance	Astra Tech	DIO			
Exceedance status						
Exceedance (Yes)	36 (60)^b^	3 (5)^c^	57 (95)^a^	96 (53.3)	99.241	<0.001
Exceedance (No)	24 (40)	57 (95)	3 (5)	84 (46.7)		

*Note:* Pearson’s chi‐square test; Values are presented as *n* (%), where *n* represents the number of measurements and % represents the percentage within each manufacturer.

^a–c^Manufacturers sharing the same letter are not significantly different.

Excellent reliability was observed across repeated measurements for all three manufacturers (ICC[3,1] = 0.992–0.998) (Table 8).

**Table 8:** Intra‐measurement agreement by manufacturer.

Manufacturer	ICC (95% CI)	*p*
Implance	0.993 (0.989–0.996)	<0.001
Astra Tech	0.992 (0.987–0.995)	<0.001
DIO	0.998 (0.997–0.999)	<0.001

Abbreviation: ICC (95% CI), intraclass correlation coefficient (95% confidence interval).

## 4. Discussion

This study evaluated the accuracy of spring‐style MTLDs under simulated clinical‐use conditions using implants inserted into CAD/CAM‐fabricated bone‐simulating resin blocks. This setup allowed each device to be tested with the corresponding implant system under standardized mechanical loading and sterilization cycles. The main finding was that torque accuracy differed markedly among manufacturers.

*A* ±10% deviation from the target torque value has commonly been used in previous MTLD studies as a threshold for acceptable accuracy [6, 10, 25, 32]. In the present study, the overall median percentage deviation was 3.33% for Astra Tech, 11.43% for Implance, and 16.67% for DIO. The manufacturer‐related difference was also reflected in the tolerance‐exceedance analysis, where DIO and Implance showed substantially higher rates of measurements outside the ±10% range than Astra Tech. These findings are consistent with Vallée et al. [8], who reported that MTLD accuracy may differ according to the device design and manufacturer. Similar variability has also been reported in studies evaluating torque‐limiting devices under clinical service or different testing conditions [10, 25]. These findings suggest that spring‐style MTLD design alone does not ensure equivalent torque accuracy across manufacturers. Verification should be performed for the specific MTLD–implant system combination used clinically.

McCracken et al. [10] found no significant difference in mean torque output between spring‐style and toggle‐style devices; however, variability was greater in the toggle‐style group. Because their study evaluated torque wrenches used in routine clinical practice, the number of sterilization cycles and frequency of use were unknown. Cehreli et al. [12] reported that manual torque devices showed a slight decrease in torque output as a result of clinical use. Mahshid et al. [13] showed that prior to steam sterilization, all tested spring‐type MTLDs remained within ±10% of the target torque value. These findings indicate that changes in torque output may occur after use or sterilization but that their direction and percentage vary according to device type and study protocol. In the present study, experimental cycles combining mechanical fatigue and sterilization affected the torque difference in all three manufacturers, although the pattern of change was not uniform. The progressive reduction in the torque difference observed in the Astra Tech group was unexpected. The reason for this pattern is unclear, and further material analyses would be needed to determine whether it reflects the mechanical adaptation of the spring mechanism after initial use.

Torque accuracy also varied according to the tested target torque value, but this finding was not observed uniformly across manufacturers. Torque difference values differed significantly between target torque settings in Astra Tech and DIO, whereas no statistically significant difference was observed between the tested settings in Implance. These findings indicate that accuracy at one torque level may not fully represent device behavior at other clinically used torque values. Verification protocols should therefore include the relevant torque settings used for both implant placement and prosthetic screw tightening.

Manufacturer‐recommended torque values should not be regarded as interchangeable fixed standards across implant systems, but rather as system‐specific reference values related to the surgical and prosthetic components of each system. In the present study, the Astra Tech [33] group showed the lowest overall deviation, remaining within the ±10% tolerance threshold at the recommended implant placement and abutment screw tightening levels, whereas the DIO [34] and Implance [35] groups exceeded this limit. This finding is relevant for DIO, because the manufacturer documentation specifies prosthetic tightening torques of 20 Ncm for the N platform and 30–35 Ncm for the R/W platforms, while fixture placement torque is listed separately as 35 Ncm. The deviation observed at the 35 Ncm setting suggests that manufacturer‐recommended torque values should still be verified with the corresponding MTLD and implant system. For Implance, the absence of clearly component‐specific torque recommendations in publicly accessible documentation limits interpretation of the observed deviation [35]. Overall, these findings support system‐specific verification of MTLD performance at the torque settings used for implant placement and prosthetic screw tightening. Accurate torque delivery is important during both implant placement and prosthetic screw tightening. During placement, insertion torque is commonly used as an indicator of primary stability; however, excessive torque may increase peri‐implant compressive stress and has been associated with marginal bone level changes [36]. In the prosthetic phase, the applied torque determines the preload within the implant‐abutment screw joint, and deviation from the intended torque may contribute to screw loosening, preload loss, component damage, or screw fracture [21]. These findings should be interpreted cautiously, because the present in vitro study did not directly evaluate clinical failure or biological outcomes.

The significant interaction between experimental cycle and manufacturer for percentage deviation shows that repeated use affected the MTLDs differently across manufacturers. For spring‐style MTLDs, this may reflect differences in device design as well as handling during torque application. Because these devices require the operator to apply force while visually monitoring the scale, torque delivery may be influenced by scale reading and operator technique. Shiba et al. [9] showed that viewing angle can affect torque accuracy and repeatability in beam‐type implant torque wrenches, and Al‐Otaibi [37] reported discrepancies between intended and achieved torque values in a simulated clinical setting.

This study has several limitations. The number of independent MTLDs was limited to three devices per manufacturer. Therefore, while the repeated‐measures design provided detailed information on within‐device changes over experimental cycles, the manufacturer‐related findings should be confirmed in studies including a larger number of MTLDs. All torque applications were performed by a single investigator, improving standardization but limiting generalizability to other operators. Because the experimental protocol combined mechanical loading with autoclave sterilization, this study could not separately evaluate the individual effects of mechanical loading and sterilization on torque accuracy. Because no material, microscopic, or deformation analysis was performed, the mechanism or onset of spring deformation could not be determined. Finally, the in vitro model could not fully reproduce clinical conditions such as restricted access, angulation, humidity, temperature, biological contamination, or patient‐specific bone characteristics.

## 5. Conclusion

Within the limitations of this in vitro study, the torque accuracy of spring‐style MTLDs differed among the tested manufacturers and changed during repeated mechanical loading and sterilization cycles. Astra Tech had the lowest overall deviation, with only a small proportion of measurements outside the ±10% range. In contrast, measurements outside this range were more frequent in the DIO and Implance groups. The influence of target torque value also differed among manufacturers, indicating that accuracy at one torque value may not reflect performance at another value.

These results suggest that spring‐style MTLDs should be checked at the torque values used for the corresponding implant system, both for implant placement and abutment screw tightening. More explicit manufacturer information on torque values for these clinical steps, as well as calibration intervals and expected limits for use or sterilization cycles, would make such deviations easier to interpret clinically. Because only three devices per manufacturer were tested under in vitro conditions, the findings should be interpreted with caution and confirmed in studies including more independent MTLDs and additional implant systems.

## Author Contributions

Conceptualization: Batuhan Aydın, Zehra Gülerol Aydın, and Serkan Polat. Formal analysis: Batuhan Aydın, Zehra Gülerol Aydın, and Serkan Polat. Investigation: Batuhan Aydın, Zehra Gülerol Aydın, and Serkan Polat. Methodology: Batuhan Aydın, Zehra Gülerol Aydın, and Serkan Polat. Writing – original draft: Batuhan Aydın, Zehra Gülerol Aydın. Writing – review and editing: Batuhan Aydın, Zehra Gülerol Aydın, Serkan Polat.

## Funding

This research received no funding.

## Disclosure

All authors read and approved of the final manuscript.

## Ethics Statement

This study was approved by the Non‐Interventional Clinical Research Ethics Committee of Bolu Abant İzzet Baysal University (Approval No. 2025/499; December 2, 2025).

## Consent

The author has nothing to report.

## Conflicts of Interest

The authors declare no conflicts of interest.

## Data Availability

The data that support the findings of this study are available from the corresponding author upon reasonable request.
